# Antiseptic Treatment of Wounds

**Published:** 1891-07

**Authors:** H. J. Warmuth

**Affiliations:** Smyrna, Tenn.


ARTICLE VI.

### ANTISEPTIC TREATMENT OF WOUNDS.

BY H. J. WARMUTH, M. D., SMYRNA, TENN.

[Read by title before the Tennessee State Medical Society, April 14, 1891.

Nothing delighted the sight of the old-time surgeons so much as the appearance of healthy pus in wounds, while to-day we endeavor not only to prevent suppuration, but at the same time to reduce all wounds, as it were, to a condition of subcutaneous injuries, in order to induce healing by primary union.

We are taught that suppuration is only various forms of fermentation, or of bacterial growth, and that wound fever, erysipelas, pyæmia, etc., are not accidents, or owing to a vitiated constitution,but are due to germs imported into wounds from without. Antisepsis is the gospel of absolute cleanliness, hence antiseptic surgery requires that anything coming in contact with a wound, should be perfectly free from germs, therefore the surgeon's hands, especially his finger-nails, instruments, sponges, dressings, etc., should be aseptic, in order to keep wounds free from suppuration.

There are many in the ranks of the profession to-day who are skeptics, or do not pay any regard to the antiseptic management of wounds, either from ignorance of the details or from a desire to follow the lead of a few eminent surgeons, We must not forget, however, that Lawson Tait is one of the quickest and most successful operators on record, and that in all his operations, wound surfaces are exposed to the action of the air but a minimum of time, and while he discards all antiseptics, yet he insists upon the most scrupulous cleanliness in his own behalf, and also on the part of his assistants and nurses, before, during and after each operation. A disregard of antiseptic principles has been productive of very serious consequences to patient and surgeon. Suppose we were to perform an amputation, and dress the stump according to ancient usage? Removing the first dressing we will probably find suppuration, afterwards, perhaps, sloughing. After a tedious seige the patient recovers and consults a lawyer. The result will be a suit for malpractice. The Court will hold, that wounds under antiseptic management will heal without suppuration, hence a disregard of this system renders us guilty of wilful negligence, and we are therefore liable for damages.

In the treatment of wounds it is necessary to adopt some kind of a plan, in order to procure the best results, and while it is not necessary to use the cumbersome Lister spray, or to use so many different antiseptics, it is desirable to have a few on hand, in order to meet any cases of emergency. A few antiseptic tablets, antiseptic powders, a bottle of carbolic acid and iodoform, are really the only antiseptics needed. In addition we need aseptic catgut or iron dyed silk for ligatures or satures, absorbent cotton, antiseptic gauze, rubber adhesive, assorted tubes for drainage and a few roller bandages. The antiseptic tablets

(Sharp and Dohme) are composed of bichloride of mercury 7.3, and ammon-chloride 7.7. Dissolved in a pint of water we have a solution of 1-1000 for cleaning the skin before operating. For small sores we use 1-3000, for larger wounds, 1-10,000, and for large joints, or the peritoneal cavity, 1-21,000. This is the most powerful germicide known. Antiseptic powders are composed of boracic acid, gr. jss, and salicylic acid, gr. xvj each. Dissolved in a pint of warm water, we have Thiersch's solution, which is milder than either the bichloride or carbolic solution. The rule is, the less irritation consistent with a thorough germicidal treatment, the better will be the chance of primary union.

Carbolic acid is also an effective germicide in 1-500. For surgical purposes we use 1-30 or 1-40. Iodoform is used as a dressing.

The application of antisepsis to a proper management of wounds depend upon wounds themselves. Small incised wounds require first, to check the hæmorrhage. If the vessels are of small size, torsion will suffice; if of larger size a ligature will be needed. Should hæmorrhage, however, have ceased, then we proceed to cleanse the surface by allowing a gentle stream of warm Thiersch's, or a weak carbolic, solution to fall upon the wound, either from a sponge or a fountain syringe. Particles of dirt or sand may be removed with forceps or a small pledget of absorbent cotton, previously dipped in an antiseptic solution. After thoroughly cleansing the wound, we dry the same with absorbent cotton, approximate the edges closely, and introduce our stitches. In this kind of wounds we do not anticipate suppuration, therefore drainage will not be necessary. After the surface has been dried, we dust iodoform all over the line of incision and wash off the excess. In a few minutes this line is covered by a crust of iodoform, which is insoluble in water. No other dressing is needed. In a few days raise the edge of the crust, cut your stitches, remove the crust, and your wound is healed. In amputations or other large operations, however, it requires three basinsfull of bichloride solution; one for sponges, one for ligatures and sutures, and the third for the purpose of continued irrigation, before, during and after the operation. Instruments require a carbolic solution 1-40 to render them aseptic. After drying the surfaces and introducing the necessary stitches, dust iodoform all over the line of incision, and begin to dress the stump by applying a silk protective over the wound, and as long as it remains green the wound is aseptic, and as soon as it changes to black it has become septic. Most surgeons, however, have discarded the protective and proceed to supply 5 to 8 layers of antiseptic gauze moistened with the antiseptic solution. The gauze then is covered carefully by a piece of rubber tissue, thoroughly immersed and disinfected in the bichloride solution. Some prefer either oiled silk or mackintosh cloth, but anyway the tissue or any of them should overlap the gauze by several inches on all sides. The whole is held in position by a few turns of a roller bandage, and this dressing may be left untouched for several days. The thermometer forms a very valuable guide as to the time of changing the dressing. A rise of temperature of 101° is an indication to change, whether the patient complains or not. After the first dressing has been removed, we proceed to thoroughly irrigate with the antiseptic solution as before, and to apply another dressing similar to the first. Where we expect more or less necrosis, however, we must provide for its removal by means of aseptic drainage tubes. They should be brought out at the most dependent part; holes are cut in the strip of antiseptic gauze for the ends of the drainage tubes to pass through. When to remove these tubes depends upon circumstances, from a few hours to three or four days and even longer.

While the above named antiseptics will prevent suppuration, we possess another agent, which will not only sterilize a suppurating wound but will also stop suppuration abruptly. Peroxide of hydrogen ($H_2O_2$) will pursue pus through the most tortuous passage into the remotest recesses and destroy pyogenic and other micro-organisms. I have employed a 15 vol. solution (Mallinckrodt, St. Louis) in carbuncles, bone felons, etc., very successfully by injecting the cavities with a small glass or rubber syringe, waiting until the foaming ceases and injecting again until the solution fails to bubble. I then apply an antiseptic dressing. The peroxide has been used by others with success in abscesses of the brain and breast, ozæna, gonorrhœa and other suppurating cavities. A close contact with metals, however, will decompose the peroxide.

I sincerely trust that the short method of treating wounds antiseptically, described in this paper, will be found not only easy of application, but will, also, by observing results, prove efficient in the management of wounds.—*Medical and Surgical Reporter.*

